# Benefit-Risk Reporting for FDA-Cleared Artificial Intelligence−Enabled Medical Devices

**DOI:** 10.1001/jamahealthforum.2025.3351

**Published:** 2025-09-26

**Authors:** John C. Lin, Bhav Jain, Jay M. Iyer, Ishan Rola, Anusha R. Srinivasan, Chaerim Kang, Heta Patel, Ravi B. Parikh

**Affiliations:** 1 Department of Medicine, Perelman School of Medicine, University of Pennsylvania, Philadelphia; 2 Department of Medicine, Stanford University School of Medicine, Stanford, California; 3 Departments of Molecular and Cellular Biology and Statistics, Harvard University, Cambridge, Massachusetts; 4 The Ohio State University College of Medicine, Columbus; 5 Division of Biology and Medicine, Brown University, Providence, Rhode Island; 6 Emory School of Medicine, Atlanta, Georgia; 7 Winship Cancer Institute, Emory University, Atlanta, Georgia

## Abstract

**Importance:**

Devices enabled by artificial intelligence (AI) and machine learning (ML) are increasingly used in clinical settings, but there are concerns regarding benefit-risk assessment and surveillance by the US Food and Drug Administration (FDA).

**Objective:**

To characterize pre- and postmarket efficacy, safety, and risk assessment reporting for FDA-cleared AI/ML devices.

**Design and Setting:**

This was a cross-sectional study using linked data from FDA decision summaries and approvals databases, the FDA Manufacturer and User Facility Device Experience Database, and the FDA Medical Device Recalls Database for all AI/ML devices cleared by the FDA from September 1995 to July 2023. Data were analyzed from October to November 2024.

**Main Outcomes and Measures:**

AI/ML reporting of study design, data availability, efficacy, safety, bias assessments, adverse events, device recalls, and risk classification.

**Results:**

The analysis included data for all 691 AI/ML devices that received FDA clearance through 2023, with 254 (36.8%) cleared in or after 2021. Device summaries often failed to report study designs (323 [46.7%]), training sample size (368 [53.3%]), and/or demographic information (660 [95.5%]). Only 6 devices (1.6%) reported data from randomized clinical trials and 53 (7.7%) from prospective studies. Few premarket summaries contained data published in peer-reviewed journals (272 [39.4%]) or provided statistical or clinical performance, including sensitivity (166 [24.0%]), specificity (152 [22.0%]), and/or patient outcomes (3 [<1%]). Some devices reported safety assessments (195 [28.2%]), adherence to international safety standards (344 [49.8%]), and/or risks to health (42 [6.1%]). In all, 489 adverse events were reported involving 36 (5.2%) devices, including 458 malfunctions, 30 injuries, and 1 death. A total of 40 devices (5.8%) were recalled 113 times, primarily due to software issues.

**Conclusions and Relevance:**

This cross-sectional study suggests that despite increasing clearance of AI/ML devices, standardized efficacy, safety, and risk assessment by the FDA are lacking. Dedicated regulatory pathways and postmarket surveillance of AI/ML safety events may address these challenges.

## Key Points

**Question:**

How comprehensively are efficacy, safety, and risk assessments reported for artificial intelligence (AI)/machine learning (ML)−enabled devices cleared by the US Food and Drug Administration (FDA)?

**Findings:**

This cross-sectional study of 691 FDA-cleared AI/ML devices found that key elements were frequently unreported, including study design (323 devices [46.7%]), training sample size (385 [53.3%]), and demographic representation (660 [95.5%]), with only 6 devices (1.6%) reporting data from randomized clinical trials, and 3 (<1%) reporting patient outcomes. Premarket safety assessments were documented for 195 devices (28.2%) and postmarket adverse events, including 1 death, were reported for 36 devices (5.2%).

**Meaning:**

These findings indicate that standardized efficacy, safety, and risk assessment reporting remains inadequate for FDA-cleared AI/ML devices, underscoring the need for dedicated regulatory pathways and robust postmarket surveillance to ensure patient safety.

## Teaser

This cross-sectional study assesses pre- and postmarket reporting of efficacy, safety, and risk for artificial intelligence−/machine learning−enabled medical devices cleared by the US Food and Drug Administration.

## Introduction

Medical devices enabled by artificial intelligence (AI) and machine learning (ML) are increasingly available for diagnosis and management in clinical areas including cancer, cardiology, and neurology.^1,2^ Both the US Food and Drug Administration (FDA) and European Union (EU) grant authorization or European Conformity marks, respectively, to allow marketing of medical devices,^3^ including AI/ML devices.^4^ As of August 7, 2024, a total of 950 AI/ML devices have been granted clearance or approval by the FDA for clinical use, with 108 and 107 cleared in 2023 and 2024, respectively.^5^

The FDA and EU classify medical devices, including AI/ML devices, according to risk.^6^ In the US, the FDA uses 3 clearance pathways for devices. (1) De novo premarket review is intended for new low- to moderate-risk (classes I or II) devices not previously classified. (2) The 510(k) clearance is intended for devices demonstrating substantial equivalence to a legally marketed predicate device.^7^ However, the 510(k) process has limitations because it does not require independent clinical evidence for every new device, relying instead on comparisons to predicate devices, which may themselves have been cleared without rigorous clinical testing, potentially allowing incremental risks to accumulate over generations of devices.^7^ (3) The Premarket Approval (PMA) pathway is the most stringent and is typically required for devices posing a substantial risk of injury or illness (class III). Most AI/ML devices are designated class II (moderate) risk and are regulated through the 510(k) pathway, which has fewer safety reporting and postmarket surveillance requirements than the PMA pathway.^7,8^ Additionally, one-third of AI/ML devices cleared through the 510(k) pathway originate from non-AI/ML devices.^9^

Unlike standards for pharmaceutical agents, there do not exist predefined standards for efficacy, safety, and risk reporting of AI/ML devices prior to or after clearance or certification.^7^ In particular, the FDA and EU have recognized safety and risk assessment as important regulatory priorities for AI/ML.^10,11^ In January 2025, the US White House issued an executive order to repeal AI policies seen as barriers to innovation, particularly a prior executive order that introduced transparency requirements for AI/ML companies, although there were no immediate impacts on AI/ML medical devices.^12^ By contrast, the EU Parliament passed the Artificial Intelligence Act^10^ in March 2024 that included safety requirements that companies must meet for AI/ML models.

To our knowledge, there has not yet been a comprehensive analysis of both pre- and postmarket benefit-risk reporting for AI/ML devices cleared by the FDA as of July 2023. Through analyzing FDA decision summaries, device labels, adverse events, and recalls, we aimed to describe benefit-risk reports, including efficacy, safety, and bias assessments, submitted by manufacturers to the FDA and to analyze postmarket surveillance of adverse events and recalls.

## Methods

The University of Pennsylvania Institutional Review Board determined that this study was exempt from review due to it being nonhuman subjects research; informed consent was likewise waived. This study followed the Strengthening the Reporting of Observational Studies in Epidemiology (STROBE) reporting guideline.

### Data Collection and Selection Criteria

We used a publicly available FDA website to identify all AI/ML devices cleared by the FDA between September 29, 1995, and July 27, 2023.^5^ The website regularly updates lists of AI/ML-enabled clearances that meet the FDA definition of medical device, including SaMD (software as a medical device) and relevant clinical decision-making software. Devices were determined to incorporate AI/ML based on information provided in market authorization summaries. We linked this FDA list to the FDA 510(k), De Novo, and PMA databases between July 2023 and January 2024, obtaining decision summaries that present the scientific evidence that served as the basis for the regulatory decision as well as data on clearance dates, reviewer specialty, company, risk classification, and clearance pathway.^13^ We also linked devices to adverse events and device recalls using device identification codes, including 510(k), De Novo, and PMA numbers, through the FDA Manufacturer and User Facility Device Experience (MAUDE) database and the Medical Device Recalls database on November 3, 2024.^14,15^

### Data Abstraction

Three investigators (J.C.L., B.J., and R.B.P.) developed a codebook for device summaries encompassing data on study design, efficacy, data availability, safety reporting, and bias assessment. Six members of the research team (J.C.L., B.J., I.R., C.K., A.R.S., and H.P.) extracted data from device summaries from July 2023 to January 2024. We also analyzed the published evidence base, adverse events, and recalls for FDA-cleared devices. Disagreements were mediated by discussion between all investigators and the principal investigator (R.B.P.) at regular meetings.

#### Premarket Variables

To evaluate study design and efficacy, we classified whether retrospective or prospective data were cited in summary documents and classified the study design for data reporting as comparative, cross-sectional, case-control, cohort, software validation and verification, or randomized clinical trial (definitions are available in eTable 1 in Supplement 1).^16,17^ We identified whether decision documents mentioned statistical and clinical metrics of efficacy. Statistical metrics included positive predictive value, negative predictive value, sensitivity, specificity, area under the curve (AUC), accuracy, and calibration. Clinical metrics included patient outcomes (eg, survival), efficiency (eg, time to intervention), and cost (eg, cost-benefit analysis).

To assess data availability, we reviewed decision summaries to identify whether primary data supporting clearance were made available online. To evaluate the availability of related peer-reviewed publications, we searched MEDLINE and Web of Science using unique device names, manufacturer names, and/or institutions noted for each device. As many as 3 searches were attempted for each device. Investigators evaluated whether publications pertained to the AI/ML device and analyzed its performance.

To assess safety reporting, we ascertained from decision summaries whether manufacturers reported devices adhering to internationally recognized safety standards for electrical technologies (ie, American National Standards Institute [ANSI], International Electrotechnical Commission [IEC], Association for the Advancement of Medical Instrumentation [AAMI], National Electrical Manufacturers Association [NEMA], or International Organization for Standardization [ISO]).^13^ Then, standards organizations were classified as electrical/software, medical, or general based on areas of specialty. For health and safety risks, we searched decision summaries for any listed error or harm associated with the device, including algorithm errors, user errors, physical harm, and/or additional adverse outcomes.

#### Postmarket Variables

To assess bias and fairness reporting, we reviewed decision summaries to evaluate whether bias assessments were conducted, specifically whether performance was reported separately for subgroups defined by race and/or ethnicity, gender or sex, age, or other factors. We also analyzed the number of reported adverse events using MAUDE and classified the reason(s) for recalls using the Medical Device Recalls database as software and hardware issues, physical and patient-related harms, or other device malfunctions.

### Statistical Analysis

First, we summarized frequencies of relevant outcomes. Given the FDA 2021 recommendations for improving transparency and safety and bias monitoring, we compared characteristics of AI/ML devices cleared before and after 2021 to identify temporal trends using χ^2^ tests; *P* < .05 were considered statistically significant. Data analysis was performed from October to November 2024, using Stata, version 18.0 (StataCorp). Our analytic dataset is available online (Supplement 3).

## Results

We identified 691 medical devices cleared and listed as AI/ML devices by the FDA (Supplement 2). Characteristics of the included devices are described in Table 1. Devices were classified by the FDA as risk class II (moderate to high risk; 691 [100%]) and were cleared through the 510(k) (668 [96.7%]), De Novo (n = 20 [2.9%]), or PMA (n = 3 [<1%]) pathways.

**Table 1.** Characteristics of Artificial Intelligence/Machine Learning−Enabled Medical Devices Cleared by the US Food and Drug Administration, 1995 to 2023

Characteristic	Devices, No. (%)
Device and clearance	
Risk class II	691 (100)
Clearance pathway	
510(k)	668 (96.7)
De Novo	20 (2.9)
PMA	3 (0.4)
Clearance time	
1995-2021	437 (63.2)
2021-Present	254 (36.8)
Specialty panel	
Radiology	531 (76.9)
Cardiovascular medicine	70 (10.1)
Neurology	20 (2.9)
Hematology	15 (2.2)
Gastroenterology-urology	11 (1.6)
Other	44 (6.4)
Study components reported	368 (53.3)
Study type	
Comparative	149 (40.5)
Cross sectional	95 (25.8)
Case-control	63 (17.1)
Cohort	55 (15.0)
Randomized clinical trial	6 (1.6)
Data collection method	305 (44.1)
Retrospective	252 (82.6)
Prospective	49 (16.1)
Both	4 (1.3)
Site location(s)	127 (18.4)
Median (IQR)	4 (2-7)
Sample size	323 (46.7)
Median (IQR)	300 (135-700)
Demographic characteristics	31 (4.5)
Participants of racial or ethnic minority group	
<20%	13 (42.0)
20%-50%	9 (29.0)
>50%	9 (29.0)
Bias assessment	60 (8.7)
Sex	49 (81.7)
Age	37 (61.7)
Race and ethnicity	17 (28.3)
Body mass index	5 (8.3)
Diabetes	1 (1.7)
Skin tone	1 (1.7)
Publicly available testing or validation	79 (11.4)
Associated with peer-reviewed research	272 (39.4)
1-2	160 (58.8)
3-9	88 (32.4)
≥10	24 (8.8)
Postmarket approval	
Adverse events reported	
Yes	36 (5.2)
No	655 (94.8)
Recall(s)	
Yes	40 (5.8)
No	651 (94.2)

Abbreviation: PMA, Premarket Approval pathway.

The medical devices analyzed were classified under 17 unique medical specialty panels. Of these specialties, radiology comprised the most devices (n = 531 [76.9%]), followed by cardiovascular medicine (n = 70 [10.1%]), neurology (n = 20 [2.9%]), hematology (n = 15 [2.2%]), and gastroenterology-urology (n = 11 [1.6%]).

### Reporting of Study Design, Data Availability, and Efficacy

In total, 323 device summaries (46.7%) did not report study design. For the 368 devices (53.3%) that reported study design (eFigure 1 in Supplement 1), the most common design was comparative (n = 149 [40.5%]), followed by cross-sectional (n = 95 [25.8%]), case-control (n = 63 [17.1%]), cohort (n = 55 [15.0%]), and randomized clinical trial (n = 6 [1.6%]).

Most device summaries did not report timing of data collection (n = 386 [55.9%]), number of site locations (n = 564 [81.6%]), or sample sizes (n = 368 [53.3%]). Among 305 devices (44.1%) that reported timing of data collection, the most common was retrospective (n = 252 [82.6%]), followed by prospective (n = 49 [16.1%]) or both (n = 4 [1.3%]) (eFigure 2 in Supplement 1). There was little variability in the number of site locations included in AI/ML device assessments (median [IQR], 4 [2-7]). The median (IQR) sample size was 300 (135-700), although this ranged from 5 to 374 160 participants. Of the 31 devices (4.5%) that reported the demographic characteristics, the study population of 13 devices (41.9%) comprised less than 20% participants of racial or ethnic minority groups; 9 (29.0%) between 20% and 50%; and 9 (29.0%) more than 50%.

Most devices (612 [88.6%]) did not make data from testing or validation available online and were not associated with a peer-reviewed research publication (419 [60.6%]). Among the 272 devices (39.4%) associated with peer-reviewed research, 160 (58.8%) were associated with 1 or 2 publications, while 24 (8.8%) were associated with 10 or more publications.

Few device summaries provided most statistical or clinical outcomes, including sensitivity (166 [24.0%]), specificity (152 [22.0%]), positive predictive value (PPV; 43 [6.2%]), negative predictive value (NPV; 36 [5.2%]), AUC (43 [6.2%]), or accuracy (33 [4.8%]). Few reported efficiency (5 [<1%]) or patient outcomes, such as vital signs and overall survival (n = 3 [<1%]).

### Reporting of Safety, Risks, and Adverse Events

Manufacturers did not report the results of a safety assessment for 496 devices (71.8%) or bias assessment for 631 devices (91.3%) and did not report adherence to international safety standards for 347 devices (50.2%) (Figure). Examples of safety assessments are described in Table 2. For devices reporting compliance with safety standards, the most common standards were electrical or software (300 [43.4%]) followed by medical (182 [26.3%]). Common standards organizations included IEC (293 [42.4%]), ISO (252 [36.5%]), NEMA (153 [22.1%]), and ANSI (123 [17.8%]). Only 42 (6.1%) devices reported potential risks to health (Table 3), such as physical harm (17 [2.5%]), algorithmic errors (29 [4.2%]), and user errors (30 [4.3%]). There was an association between approval pathway (510[k], De Novo, PMA) and reporting of safety assessments (510[k], 486 of 668 [72.8%]; De Novo, 9 of 20 [45.0%]; and PM, 1 of 3 [33.3%]; *P* = .008), but not reporting of bias assessments or adverse events. In total, 489 adverse events were reported to the FDA for 36 devices (5.2%) , including 458 malfunctions, 30 injuries, and 1 death.

**Figure.**
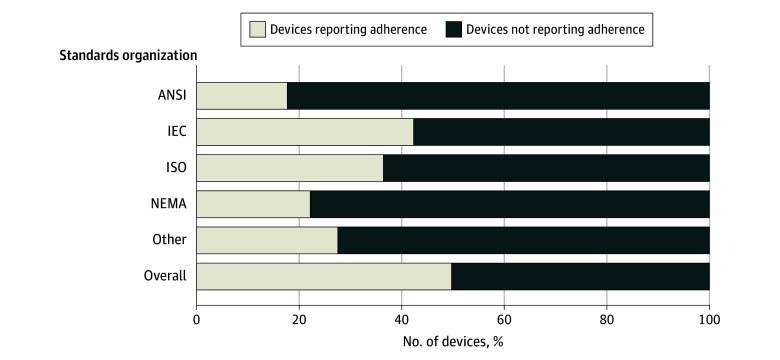
Safety Standards Reported by US Food and Drug Administration Approved Artificial Intelligence/Machine Learning−Enabled Medical Devices ANSI indicates American National Standards Institute; IEC, International Electrotechnical Commission; NEMA, National Electrical Manufacturers Association; ISO, International Organization for Standardization.

**Table 2.** Selected Summaries of Safety Assessments Performed for Artificial Intelligence/Machine Learning−Enabled Medical Devices^a^

Device name	Manufacturer	Description of safety
THINQ	CorticoMetrics	Validation included performance testing for accuracy (comparisons to expert-labeled brain images) and reproducibility (test-retest image data).
BodyGuardian Remote Monitoring System	Preventice Technologies	EMC and electrical safety testing, software verification/validation, biocompatibility for patient contact materials, performance testing, predicate device comparison tests, usability testing.
Biograph Vision, Biograph mCT family of PET/CTs	Siemens Medical Solutions	All features (resolution, count rate/scatter/sensitivity, image quality, coregistration accuracy) tested during verification/validation testing and met the predetermined acceptance criteria. Changes (OncoFreeze AI [data-driven gating], SUVmax, SUVmean, volume measurement) underwent scientific evaluation.
Hepatic VCAR	GE Medical Systems	Quality assurance measures have been applied to the development of the device: risk analysis, requirements reviews, design reviews, performance testing (verification, validation), safety testing (verification).
Accelerate Pheno System, PhenoTest BC kit	Accelerate Diagnostics	Performance established during an internal performance evaluation study, with results. Internal reproducibility study conducted according to FDA AST class II Special Controls guidance, with results. Electrical safety and EMC testing is identical to that conducted for the predicate device. Software verification and validation testing conducted and documentation provided.

Abbreviations: AI, artificial intelligence; AST, antimicrobial susceptibility test; BC, blood culture; EMC, electromagnetic compatibility; FDA, US Food and Drug Administration; PET/CT, positron emission tomography/computed tomography; SUV, standard uptake value; VCAR, volume computer assisted reading.

^a^ These excerpts and the complete descriptions of safety assessments are available from FDA (accessdata.fda.gov/scripts/cdrh/cfdocs/cfPMN/pmn.cfm).

**Table 3.** Safety Risks of Artificial Intelligence/Machine Learning−Enabled Medical Devices Reported in Decision Summaries

Safety risk	Devices, No.	Examples
Algorithm errors	29	Device failure or delay, data corruption, false positives/negatives
User errors	30	Improper dosing, device misuse, misinterpretation of results
Physical harm	17	Adverse tissue reaction, electrical shock, eye hazard or injury
Other risks	3	Data breaches due to insecure transmission of data, interference with other devices

### Device Recalls

Forty devices (5.8%) were recalled 113 times in total for reasons including software or hardware issues, physical harm to patients, or other device malfunctions (Table 4). Most recalls (85 [75.2%]) were due to software issues, such as delays in starting hardware and patient management, improper dosing of medications, and imaging scans being lost or sent to multiple patients. Some (26 [23.0%]) recalls were due to physical- or patient-related harms (eg, excessive noise that could cause hearing loss). Devices with adverse events were more likely to be recalled than those without adverse events (14 of 36 [38.9%] vs 26 of 655 [4.0%]; *P* < .001).

**Table 4.** Characteristics of Recalls of Artificial Intelligence/Machine Learning−Enabled Medical Devices

Device name	Recalls, No.	Reason for recall	Patient harm	Example of recall details
A	1	Device failure	No	Low battery life
B	1	Device failure	No	Internal failure of the vertical/horizontal tilt adjustment mechanism
C	2	Device failure	Yes	Improperly formulated and released fluorescence in situ hybridization probes resulting in false positives
D	10	Software and device failure	Yes	Complaints due to compromised hyperbaric chamber, optical degradation may potentially cause a delay in reporting results
E	1	Device failure	No	Issue with locking mechanism preventing scanning
F	2	Device failure	No	Issue with locking mechanism preventing scanning
G	4	Physical harm, software and device failure	Yes	Elevated acoustic noise during scanning, potentially leading to hearing loss
H	1	Device failure	No	Lubricating grease may cause visible, dot- or line-shaped, fat-isointense artifacts during head examinations
I	1	Device failure	No	Lubricating grease may cause visible, dot- or line-shaped, fat-isointense artifacts during head examinations
J	27	Software and device failure	No	Issue with propagation of treatment course information, software error resulting in local underestimation of expected dose, issues applying pitch/roll correction
K	1	Device failure	No	Initial delivery positions will be set incorrectly despite calibration
L	2	Device failure	No	System operator is able to bypass the SmokeDetector Interlock system locking mechanism after a smoke detection
M	8	Software and device failure	No	Potential for Incorrect Image Orientation resulting images may be flipped or reversed result in misdiagnosis, incorrect treatment of a condition, or additional radiation exposure if a rescan is required
N	9	Software and device failure	No	Dosing errors and duplication, software issues resulting in incorrect data transmission and patient care planning
O	1	Device failure	No	Probe handle may crack
P	1	Device failure	No	Potential for amplified noise and/or overall signal reduction, which may interfere with intended recordings of heart rhythms
Q	2	Software and labeling	No	Default reference ranges are incorrectly displayed, resulting in incorrect readings
R	1	Labeling	No	Missing precautionary statements
S	7	Physical harm and software	Yes	Potential for composed images to be flipped, resulting in misdiagnosis
T	1	Software	No	Low sensitivity, resulting in delays in diagnosis and treatment
U	2	Software	No	Wrong calculation of the dose/min for fluoroscopy examinations
V	6	Software	No	May bring up another patient when comparing scans
W	1	Software	No	Insufficient documentation of quality procedures
X	1	Software	No	Data transmission issues
Y	1	Software	No	Inability to boot up in timely fashion
Z	1	Software	No	Inability to boot up in timely fashion
AA	1	Software	No	Duplicate logging of a blood glucose level reading
AB	2	Software	No	Inconsistent configuration
AC	1	Software	No	Data not properly transmitted
AD	1	Software	No	Partial electrical reset results in inability to detect and report Brady and Pause events
AE	2	Software	No	Incorrect dosing or dose applied to the wrong location
AF	1	Software	No	Incorrect measurements
AG	1	Software	No	CT scan failing resulting in rescanning and reinjection of contrast medium.
AH	1	Software	No	Unintended video frames being included, resulting in incorrect estimates
AI	1	Software	No	Diagnostic imaging system failing resulting in rescanning and reinjection of contrast medium
AJ	1	Software	No	Potential for data loss
AK	1	Software	No	Potential interruption of data communication
AL	1	Software	No	Issues with terminating scan, resulting in treatment delay
AM	1	Software	No	Misleading and out-of-sync display
AN	2	Software and device failure	No	False-negative results due to reagent packs exhibiting low signal

Abbreviation: CT, computed tomography.

### Temporal Trends

Relative to FDA devices cleared before 2021, devices cleared during or after 2021 were more likely to publish data online (72 of 254 [28.4%] vs 7 of 437 [1.6%]; *P* < .001); evaluate demographic bias (49 of 254 [19.3%] vs 11 of 437 [2.5%]; *P* < .001); and report study timing (133 of 254 [52.4%] vs 172 of 437 [39.4%]; *P* < .001), efficacy (91 of 254 [35.8%] vs 104 of 437 [23.8%]; *P* < .001) and clinical (28 of 254 [11.0%] vs 5 of 437 [1.1%]; *P* < .001) outcomes. However, devices approved during or after 2021 were less likely to be associated with a peer-reviewed publication (81 of 254 [31.9%] vs 191 of 437 [43.7%]; *P* = .002), report safety assessment results (34 of 254 [13.4%] vs 161 of 437 [36.8%]; *P* < .001), and have adverse events (5 of 254 [2.0%] vs 31 of 437 [7.1%]; *P* = .003) than those approved before 2021. No differences were identified in reported study design (observational vs controlled), study timing (prospective vs retrospective), adherence to international standards, reporting of health risks, or recalls (eTable 2 in Supplement 1). Devices cleared through the 510(k) pathway before 2021 were marginally more likely to report safety and bias assessments. However, this relationship was not evident for devices approved through the same pathway during or after 2021 (eTable 2 in Supplement 1).

## Discussion

In this cross-sectional study of all FDA-cleared AI/ML devices from September 1995 to July 2023, a large proportion of decision summaries—intended to contain data supporting clearance—lacked information regarding study design, timing, efficacy, clinical outcomes, safety assessment, adherence to safety standards, demographic characteristics, bias assessment, and data availability. Most cleared devices did not have a study design or timing included in the clearance summary or in a peer-reviewed publication. Additionally, for the few devices that did report study design and timing, most were cleared based on retrospective observational studies. Some devices (39.4%) had results that were published in peer-reviewed literature. Safety risks reporting was uncommon (6.1%), usually focused on algorithmic or user errors, and did not adhere to international safety standards (ie, IEC, ISO, NEMA, or ANSI).

Our findings were consistent with previous evidence^7,8^ in an older cohort of approvals, suggesting a paucity of prospective evidence for AI/ML devices. Our study augments these findings by elucidating pre- and postmarket safety concerns, including finding that 5.2% of devices reported postmarket adverse events and 5.8% of cleared devices were recalled, with a meaningful percentage of recalls due to patient-specific harms. These rates are lower than the rates of adverse events and recalls of non-AI/ML devices submitted in the 510(k) pathway,^18,19^ such as surgical and implantable devices.^20,21,22,23^ However, this finding raises concerns about the adequacy of the 510(k) framework for evaluating substantial equivalence for medical devices, particularly AI/ML devices, as the absence of robust clinical validation may limit the ability to assess performance and patient impact in the community setting.^18,19^ Existing FDA postmarket surveillance databases documented 489 adverse events for 36 devices and 111 recalls of 39 devices; devices with adverse events were more likely to be recalled.

Radiology and cardiovascular medicine were the most common specialties for devices. Medical device applications are designated to 1 of 19 FDA specialty panels for review; each has varying levels of workload and resources.^24^ Moreover, reporting of safety results and risks to health, as well as adherence to international standards, did not improve after 2021. These findings suggest that premarket safety information for AI/ML devices may be insufficient and more robust postmarket surveillance systems are necessary to capture health-related adverse events and safety concerns relevant to AI/ML devices. In particular, known degradation in performance of AI/ML algorithms complicates traditional surveillance methods.^25^ Nonlocked AI/ML models can self-learn based on new data acquired during patient care, but AI/ML algorithm performance often drifts over time.^26,27^ Voluntary adverse event reporting databases, such as MAUDE, may not capture eventual diagnostic or prognostic errors associated with these AI/ML performance gaps. It may be critical to establish consensus on what constitutes an AI/ML-related adverse event to implement dedicated automated mechanisms for postmarket surveillance of AI/ML devices, potentially through linkages to EHR systems.

Moreover, we find that most risks to health reported in existing surveillance systems focus on algorithmic and user errors, neglecting the need for standardized characterization of patient-level safety risks associated with AI/ML devices. These could include standardized adverse events (eg, unnecessary biopsies, missed diagnoses of pathologic specimens) that could result from faulty AI/ML devices. Furthermore, the well-documented underreporting of safety events in MAUDE necessitates a shift toward more proactive safety monitoring.^28^ Leveraging end user health systems to report safety events and performance drift of FDA-cleared devices could provide a more comprehensive and timely understanding of how AI may affect patient outcomes. In 2021, the FDA called for improving transparency, monitoring safety and bias, and ensuring test-data quality of AI/ML devices.^7^ We found that even when bias and safety were evaluated, results were typically not disclosed to the public.

AI/ML devices cleared after 2021 were mostly reliant on retrospective observational studies.^7^ These findings add to prior evidence noting overwhelmingly nonprospective data underlying AI/ML device clearances. Our study adds to prior literature by characterizing temporal trends in evidence quality for AI/ML devices. There were some improvements over time in transparency: post-2021 devices were substantially more likely to report study timing (52.4% post-2021 vs 39.4% pre-2021), efficacy (35.8% vs 23.8%), and clinical outcomes (11.0% vs 1.1%). Based on its pathway, devices cleared before 2021 varied in their reporting of safety and bias assessments—this association was not observed in devices cleared during 2021 and after. However, devices cleared after 2021 were also less likely to be associated with peer-reviewed research. Overall, greater standardization of transparent outcome reporting is essential for premarket clearance of AI/ML devices.

Our findings also show a pressing need to center health equity in the development of AI/ML devices. Most devices did not report the study population’s demographic characteristics (95.5%) and bias assessment (91.3%) for the patients in whom their algorithms were tested. Among the few that did report demographic information, individuals of racial or ethnic minority groups were underrepresented. This imbalance was further exacerbated by the limited scope of bias assessments conducted, which primarily focus on sex and age. Critical factors such as race and ethnicity, body mass index, and skin tone were often not reported. Lacking these comprehensive evaluations can lead to AI/ML devices inadvertently embedding and amplifying existing biases in clinical care.^29^ Although reporting of bias calculations improved after 2020, in public reports and documents 80.7% of devices still lacked relevant demographic information about their training or testing cohort.

### Limitations

Our study has several limitations worth noting. First, while decision summaries aim to provide a comprehensive overview of scientific data, they may not always contain all necessary information submitted to the FDA for clinical use.^13^ However, most efficacy and safety data are expected to be included in these summaries and should promote public transparency. Second, additional safety assessments may occur after FDA clearance, meaning the full scope of information essential for clinical use may not always be available in premarket decision summaries. However, we extensively interrogated adverse event and recall databases to capture potential safety issues attributable to these devices. Third, devices may receive multiple FDA clearances over time, and our analysis was conducted at the level of individual clearances, which may not capture all prior or subsequent regulatory decisions. In addition, certain data, such as the number of peer-reviewed publications, can be challenging to link to specific devices due to differences between the submitted and published versions of a device name or type. To attempt to address this, we conducted at least 2 independent reviews of each publication to verify its relationship with each device.

Several key considerations warrant further attention. First, the Instructions for Use for each device did not yield relevant information for our analysis. Second, the low number of reported adverse events raises concerns about the adequacy of postmarket surveillance. Our statistical analyses underscore the need for more robust reporting mechanisms to ensure accurate postmarket safety assessments. Lastly, our study did not differentiate between locked and generative AI in the regulatory approval process due to an inadequate sample of generative AI devices (2 of 691 [0.3%]). Future research should explore whether the clearance pathways differ between these AI types and how regulatory oversight adapts to the unique risks and challenges posed by generative AI.

## Conclusions

This cross-sectional study found that improved premarket data transparency and rigorous evaluation of AI/ML device efficacy and safety are needed. Using decision summaries for FDA-cleared AI/ML devices linked to adverse event and recall data, we found that currently, devices lack transparent efficacy or safety evaluations despite postmarket safety events being observed. To date, the FDA does not have a dedicated regulatory pathway for AI/ML devices.^4^ Given the unique considerations of these devices, implementation of a dedicated regulatory system for AI/ML devices, including more rigorous standards for study design and transparency, should be considered. Additionally, a more rigorous postmarket monitoring system may facilitate trust in integrating AI/ML devices into clinical settings.

## Notes

Supplement 1. eTable 1. Classification System for Study Designs for AI/ML Devices eTable 2. Differences in Characteristics Between Artificial 1 Intelligence/Machine Learning Devices Cleared by the US Food and Drug 2 Administration Over Time, 1995 to 2020 vs 2021 to 2023 eFigure 1. Trends in Study Design of FDA-Cleared AI/ML Devices, 1995-2023 eFigure 2. Trends in Timing of Data Collection of FDA-Cleared AI/ML Devices, 1995-2023

Supplement 2. Characteristics of FDA-Cleared Artificial Intelligence/Machine Learning-Enabled Medical Devices, 1995-2023

Supplement 3. Data Sharing Statement
