# Supplementary material for: Benefit-Risk Reporting for FDA-Cleared Artificial Intelligence−Enabled Medical Devices
Source: JAMA Health Forum. 2025 Sep 26;6(9):e253351. doi: 10.1001/jamahealthforum.2025.3351 (PMC12475944; doi:10.1001/jamahealthforum.2025.3351)
Supplement: Supplement 1. — eTable 1. Classification System for Study Designs for AI/ML Devices eTable 2. Differences in Characteristics Between Artificial 1 Intelligence/Machine Learning Devices Cleared by the US Food and Drug 2 Administration Over Time, 1995 to 2020 vs 2021 to 2023 eFigure 1. Trends in Study Design of FDA-Cleared AI/ML Devices, 1995-2023 eFigure 2. Trends in Timing of Data Collection of FDA-Cleared AI/ML Devices, 1995-2023 [file jamahealthforum-e253351-s001.pdf]

Supplemental Online Content

Lin JC, Jain B, Iyer JM, et al. Benefit-risk reporting for FDA-cleared artificial intelligence−enabled medical devices. *JAMA Health Forum.* Published online September 26, 2025. doi:10.1001/jamahealthforum.2025.3351

eTable 1. Classification System for Study Designs for AI/ML Devices

eTable 2. Differences in Characteristics Between Artificial 1 Intelligence/Machine Learning Devices Cleared by the US Food and Drug 2 Administration Over Time, 1995 to 2020 vs 2021 to 2023

eFigure 1. Trends in Study Design of FDA-Cleared AI/ML Devices, 1995-2023

eFigure 2. Trends in Timing of Data Collection of FDA-Cleared AI/ML Devices, 1995-2023

This supplemental material has been provided by the authors to give readers additional information about their work.

© 2025 Lin JC et al. *JAMA Health Forum*.

eTable 1: Classification System for Study Designs for AI/ML Devices

Study Design	Description
Comparative	Studies that directly compare two or more similar groups to analyze differences in outcomes or effects.
Cohort	Studies that follow a group of individuals over a period of time to observe the development of certain outcomes based on exposures or interventions.
Case-Control	Studies that compare individuals with a particular condition (cases) to those without the condition (controls) to identify potential factors associated with the condition.
Cross-Sectional	Studies that analyze data collected from a population at a single point in time to examine relationships between variables or characteristics.
Software Validation	Process of ensuring that software meets specified requirements and functions correctly according to its intended purpose.
RCTs	Randomized Controlled Trials: Studies in which participants are randomly assigned to different groups to compare the effects of treatments or interventions.

© 2025 Lin JC et al. *JAMA Health Forum*.

eTable 2: Differences in Characteristics Between Artificial 1 Intelligence/Machine Learning Devices Cleared by the US Food and Drug 2 Administration Over Time, 1995 to 2020 vs 2021 to 2023

Characteristics	Reporting, No. (%)		*P* value
	1995-2020	2021-2023	
Type of study			
Observational	132 (61.4)	81 (52.9)	.11
Controlled	83 (38.6)	72 (47.1)	
Reporting of data collection method			
Yes	172 (39.4)	133 (52.4)	<.001
No	265 (60.6)	121 (47.6)	
Method of data collection			
Prospective	32 (18.6)	17 (13.2)	.21
Retrospective	140 (81.4)	112 (86.8)	
Reporting of demographic bias assessments			
Yes	11 (2.5)	49 (19.3)	<.001
No	426 (97.5)	205 (80.7)	
Reporting of efficacy outcomes			
Yes	104 (23.8)	91 (35.8)	<.001
No	333 (76.2)	163 (64.2)	
Reporting of clinical outcomes			
Yes	5 (1.1)	28 (11.0)	<.001
No	432 (98.9)	226 (88.98)	
Reporting of safety assessment results			
Yes	161 (36.8)	34 (13.4)	<.001
No	276 (63.2)	220 (86.6)	
Adherence to international standards			
Yes	208 (47.6)	136 (53.5)	.13
No	229 (52.4)	118 (46.5)	
Reporting of health risks			
Yes	23 (5.3)	19 (7.5)	.24
No	414 (94.7)	235 (92.5)	
Device recalled			
Yes	31 (7.09)	9 (3.5)	.05
No	406 (92.91)	245 (96.5)	
Adverse events			
Yes	31 (7.1)	5 (2.0)	.003
No	406 (92.9)	249 (98.0)	
Testing/validation publicly available			
Yes	7 (1.6)	72 (28.4)	<.001
No	430 (98.4)	182 (71.7)	
Link to peer-reviewed research			
Yes	191 (43.7)	81 (31.9)	.002
No	246 (56.3)	173 (68.1)	

Abbreviations: AI/ML, artificial intelligence and machine learning; FDA, US Food and Drug Administration.

© 2025 Lin JC et al. *JAMA Health Forum*.

eFigure 1: Trends in Study Design of FDA-Cleared AI/ML Devices, 1995-2023

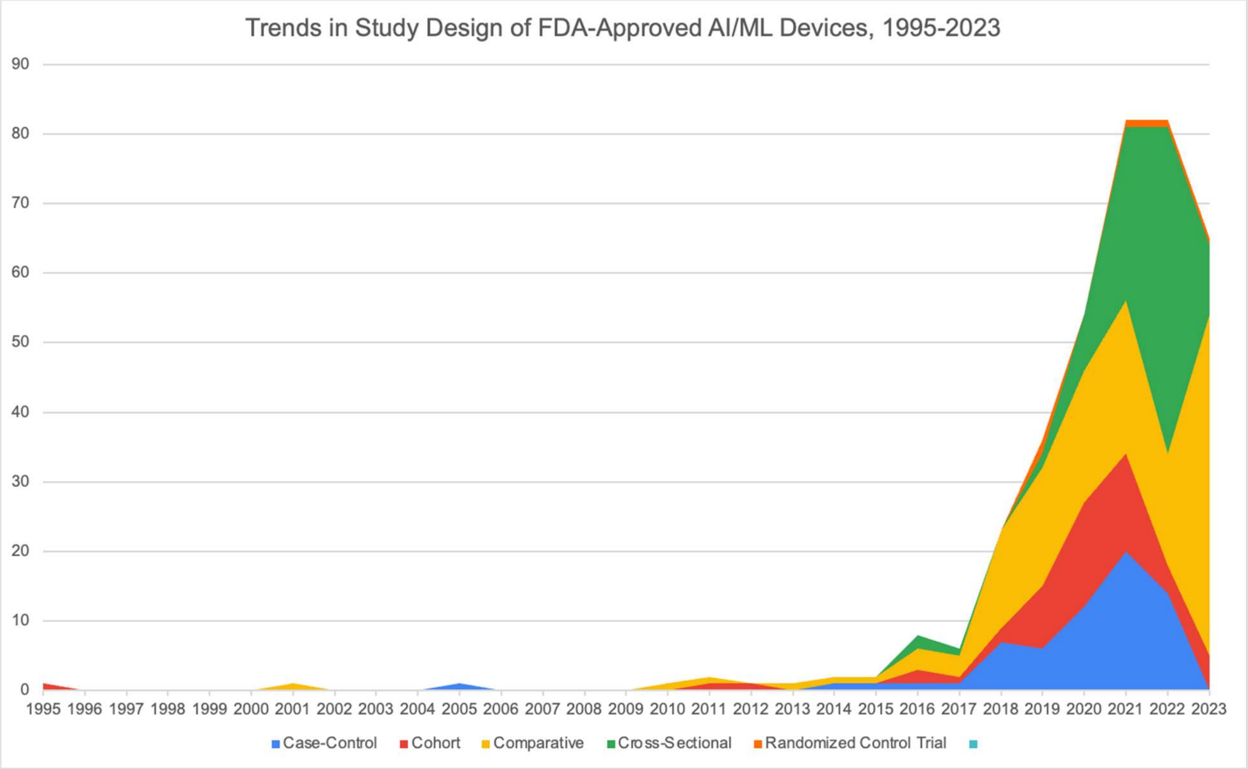

AI/ML, artificial intelligence/machine learning; FDA, Food and Drug Administration.

© 2025 Lin JC et al. *JAMA Health Forum*.

eFigure 2: Trends in Timing of Data Collection of FDA-Cleared AI/ML Devices, 1995-2023

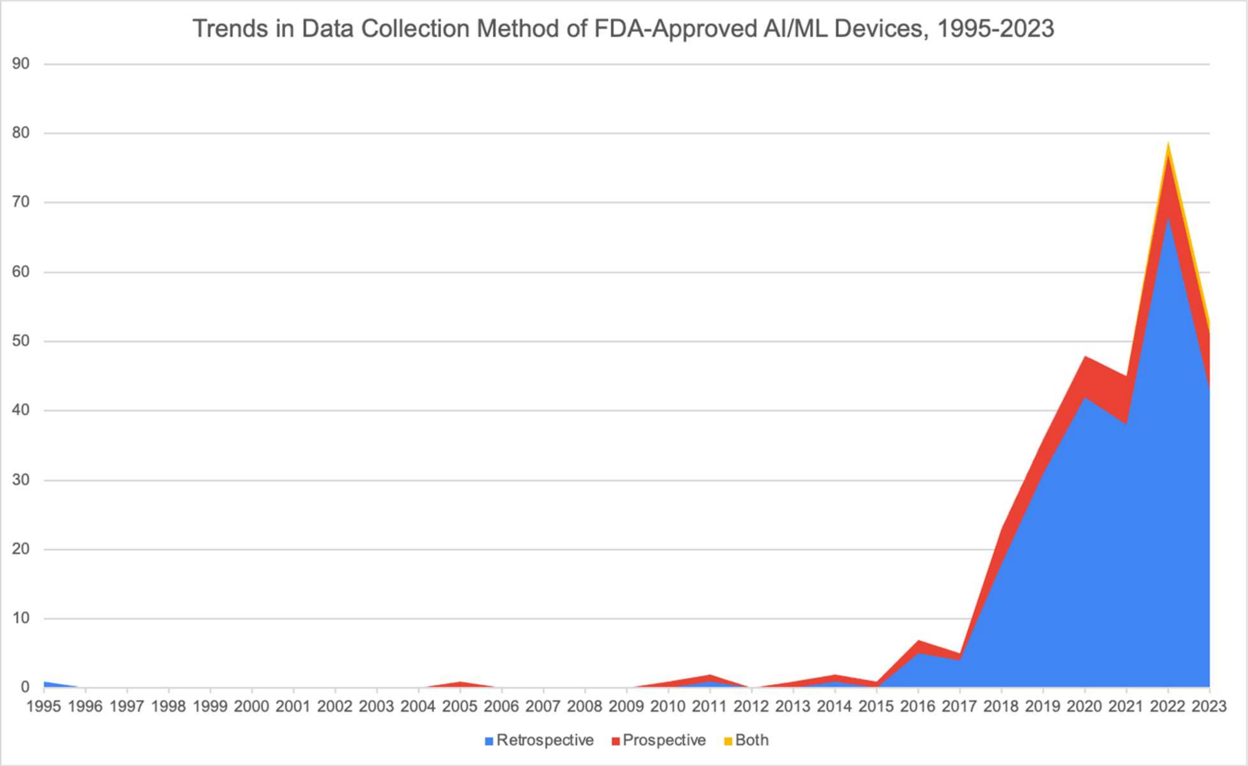

AI/ML, artificial intelligence/machine learning; FDA, Food and Drug Administration

© 2025 Lin JC et al. *JAMA Health Forum*.
