# Supplementary material for: Benefit-Risk Reporting for FDA-Cleared Artificial Intelligence−Enabled Medical Devices
Source: JAMA Health Forum. 2025 Sep 26;6(9):e253351. doi: 10.1001/jamahealthforum.2025.3351 (PMC12475944; doi:10.1001/jamahealthforum.2025.3351)
Supplement: Supplement 3. — Data Sharing Statement [file jamahealthforum-e253351-s003.pdf]

Data Sharing Statement

Lin. Benefit-Risk Reporting for FDA-Cleared Artificial Intelligence–Enabled Medical Devices. *JAMA Health Forum*. Published September 26, 2025. doi:10.1001/jamahealthforum.2025.3351

Data
Data available: Yes
Data types: Data (not involving human participants)
How to access data: https://github.com/HumanAlgorithmCollaborationLab/AI-ML-Regulation
When available: With publication

Supporting Documents
Document types: Statistical/analytic code
How to access documents: All data and documentation are included in this submission.
When available: With publication

Additional Information
Who can access the data: All data are available online for any purpose with investigator support.
Types of analyses: All data are available online for any purpose with investigator support.
Mechanisms of data availability: All data are available online for any purpose with investigator support.
